# State of the Art in Novel Treatment Strategies in Rheumatoid Arthritis: A Brief Review

**DOI:** 10.31138/mjr.241124.ath

**Published:** 2025-06-30

**Authors:** Melissa Macedo Peixoto Nascimento, Alef Azuelos, Ivna Lacerda Pereira Nóbrega, Milena Sales Pitombeira, Ana Tereza Amoedo Martinez, Jozélio Freire de Carvalho, Carlos Ewerton Maia Rodrigues

**Affiliations:** 1 Medical School, Universidade de Fortaleza, Ceará, Brazil;; 2 Graduate Program in Medical Sciences, Universidade de Fortaleza (Unifor), Fortaleza, Brazil;; 3 Hospital Geral de Fortaleza, Ceará, Brazil;; 4 Novaclin, Grupo CITA< Salvador, Bahia, Brazil;; 5 Núcleo de Pesquisa em Doenças Crônicas não Transmissíveis (NUPEC), School of Nutrition from the Federal University of Bahia, Salvador, Bahia, Brazil;; 6 Department of Internal Medicine, Federal University of Ceará, Brazil

**Keywords:** rheumatoid arthritis, treatment, immunobiological

## Abstract

Rheumatoid arthritis (RA) is a chronic autoimmune inflammatory disease with substantial morbidity and socioeconomic burden. Early diagnosis and treatment are crucial to mitigate disease progression and preserve joint function. Current treatment strategies encompass non-pharmacological and pharmacological approaches, with disease-modifying antirheumatic drugs (DMARDs) being the cornerstone of pharmacotherapy. Novel immunomodulating drugs have revolutionised RA management by targeting specific cytokines or intracellular signalling pathways involved in disease pathogenesis. Evidence-based guidelines recommend biologics as second-line therapy for patients for whom conventional DMARDs have failed. While Tumour Necrosis Factor Inhibitors (TNFi) have traditionally been favoured, recent evidence suggests nuanced responses based on patient characteristics and treatment history are more effective. Moreover, Janus Kinase (JAK) Inhibitors emerge as a promising therapeutic option, demonstrating comparable efficacy to bDMARDs in clinical practice. Despite significant advancements, challenges in optimising RA treatment include variable treatment responses and safety concerns. Future research aims to refine treatment strategies, personalise therapeutic approaches, and elucidate disease mechanisms to improve outcomes for RA patients. The evolving landscape of immunomodulating drugs offers diverse therapeutic options for RA management. This article provides a comprehensive review of RA therapy, focusing on novel immunomodulating drugs.

## INTRODUCTION

Rheumatoid arthritis (RA) is a chronic systemic auto-immune inflammatory disease that affects approximately 17.6 million people worldwide, with a higher prevalence in females in high-income countries and urban settings.^1, 2^

Although the aetiology of RA remains unclear, environmental and sociodemographic characteristics, such as genetic predisposition, epigenetics, smoking, and inflammation of the mucosa, may activate immune responses preceding the development of the disease.^3^ The initial activation of the immune system leads to the infiltration of inflammatory cells, such as macrophages, T cells, and B cells, into the synovial tissue of the affected joint.^4^ Abnormalities in immune response lead to the formation of autoantibodies, such as rheumatoid factors (RF), antibodies against post-translationally modified proteins, lymphocyte infiltration into the synovium, angiogenesis, and inflammatory cytokine production.^3,5,6^

RA evolves in a fluctuant manner, with exacerbations leading to the gradual onset of additive joint pain, stiffness, and swelling affecting multiple joints, which gradually leads to loss of function and, if untreated, joint destruction with cartilage erosion and bone damage.^7,8^ Early diagnosis and treatment are fundamental for disease control and prevention of functionality loss.^9^ Current treatment pathways aim to improve the quality of life while achieving clinical remission and reducing disease activity. Different treatment strategies, including non-pharmacological and pharmacological therapies, have been used to improve outcomes. Non-pharmacological approaches aim to decrease anxiety and depression, reduce pain, and increase mobility. Pharmacological treatment is divided mainly into symptomatic treatment and disease-modifying drugs (DMARDs).^10^

Symptomatic treatment consists of glucocorticoids (GCs) and nonsteroidal anti-inflammatory drugs (NSAIDs). Disease-modifying antirheumatic drugs (DMARDs) have the goal of slowing progression and preventing complications. Conventional synthetic (cs) DMARDs like hydroxychloroquine, methotrexate (MTX), sulfasalazine, and leflunomide, are considered first-line treatment for RA. However, many patients fail to respond to these treatments, for which biological (b) DMARDs (tumour necrosis factor inhibitors, interleukin 6 receptor inhibitors, anti-CD20, and anti-CD80/86) or targeted synthetic (ts) DMARDs (Janus kinases (JAK) inhibitors) have been developed and approved for clinical use. These immunomodulating agents constitute secondary and tertiary lines and are an alternative therapeutic option for individuals who have experienced treatment failure with csDMARDs.^11,12^ The bDMARDs actions are not through direct intracellular modifications but through mediating their respective modes of action outside the cell or via the cell surface.^13^ This review describes the state of the art in RA therapy using novel immunomodulating drugs.

## SEARCH STRATEGY

**Figure 1.**
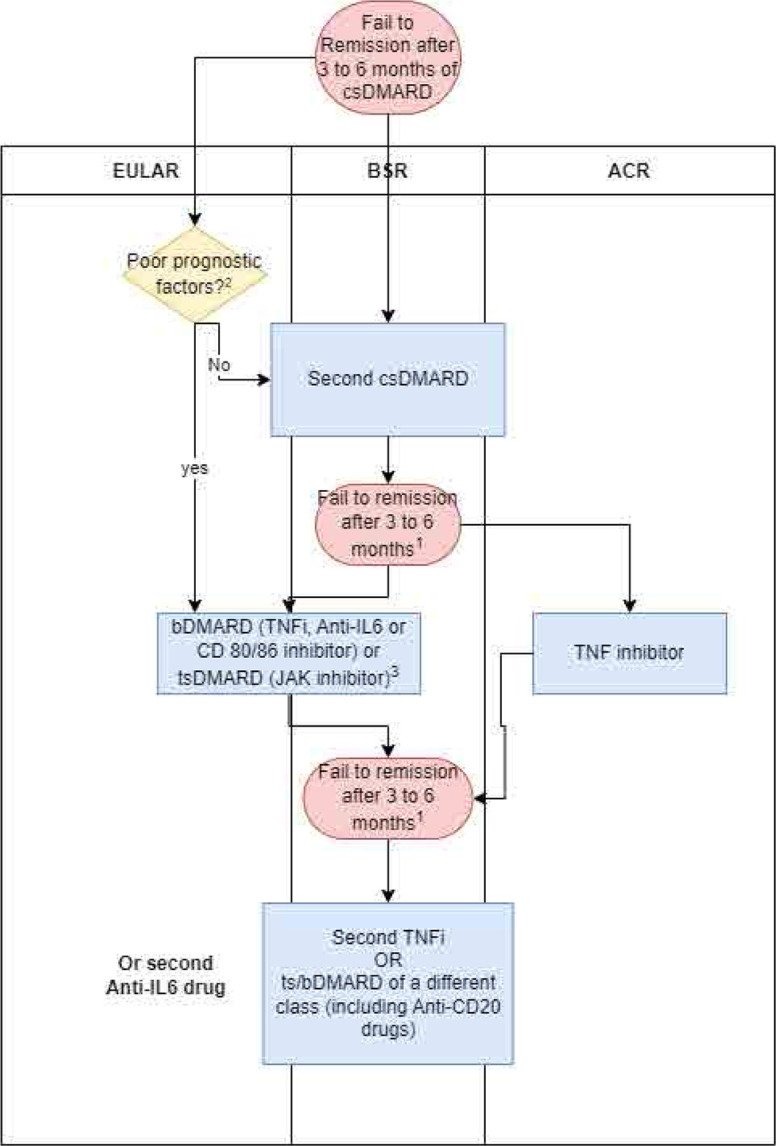
Comparative flowchart of the main treatment guidelines for rheumatoid arthritis (EULAR, ACR, and BSR). ACR: American College of Rheumatology; EULAR: European League Against Rheumatism; BSR: Brazilian Society of Rheumatology.

For this narrative review, an electronic literature search was carried out in the Medline/PubMed, Scopus, and Capes databases. The following keywords used were “rheumatoid arthritis”, “treatment”, “immunobiological”. We included clinical trials, original articles, narrative and systematic reviews and guidelines relevant to the aim of our review. There were no limits on the date of publication and publications in languages other than English or Spanish were excluded. All relevant studies up to June 2024 were included. The articles found were reviewed in relation to immunopathogenesis of RA and novel drugs, and we propose to compare the main RA guidelines about the new strategies of treatment for RA (**Figure 1**).

## IMMUNOPATHOGENESIS OF RA

RA exhibits a complex pathogenesis, with genetic, environmental, immunologic, and other factors converging to initiate and manifest the disease. Although the exact cause remains uncertain, there is an understanding that environmental and genetic elements interact, initiating adaptive responses related to autoimmunity before emergence of clinical symptoms.^14^ The initial phases are likely triggered by exposure to environmental factors, impacting mucosal surfaces; these include exposure to substances like cigarette smoke in the airway.^14^ This exposure activates peptidyl arginine deiminases (PADs), which converts arginine to citrul-line, modifying these peptides. The altered proteins are then presented to T cells via antigen-presenting cells (APCs), such as dendritic cells (DCs). While these processes may occur in mucosal regions, they are not limited to this location. They can also emerge in central lymphoid organs, producing antibodies targeting the modified peptides.^14^

Before the onset of RA symptoms, anti-citrullinated protein antibodies (ACPAs) and cytokines increase over time.^15^ Although the specific events precipitating synovitis are unclear, a likely contributor is a second “strike,” such as forming immune complexes, heightening vascular permeability in the synovium, and activating synovial cells.^15^ Subsequent involvement of small-molecule inflammatory mediators, autoantibodies and cytokines triggers processes that leads to the perpetuation of arthritis.^15^ Furthermore, synovial inflammation causes the activation of mesenchymal cells within the joint, displaying aggressive behaviour and the capacity to invade and destroy cartilage. Concurrently, osteoclasts cause damage to the subchondral bone, which leads to irreversible loss of articular cartilage and bone begins shortly after RA onset, underscoring the critical importance of early interventions.^15^

While the activation of innate immunity and the citrullination of proteins likely represent initial steps in the onset of RA, adaptive immunity assumes a pivotal role in this initiating process. Within an inflammatory environment, APCs become activated and loaded with either native or modified proteins.^16^ Subsequently, these APCs migrate to central lymphoid organs, presenting diverse antigens to T cells. These T cells, once activated, undergo differentiation into effector, memory, and regulatory T cells. Collaborating with the activated T cells, the APCs facilitate the transformation of B cells into antibody-secreting cells. Following this orchestrated immune response, activated APCs, T cells, and B cells then migrate back to the synovium, contributing to the perpetuation of the chronic inflammatory response in rheumatoid arthritis.^16^

Various cell types within the rheumatoid synovium, including T and B cells, macrophages, and fibroblast-like synoviocytes (FLS), can release cytokines. The initiation and persistence of RA are notably impacted by the communication that occurs through secretion of proinflammatory cytokines.^17^

It is essential to highlight that not all anti-cytokine therapies exhibit effective outcomes. Specific cytokines, like IL-1 beta, though significant, seem to play a relatively smaller role in the inflammatory mechanisms of RA compared to TNF and IL-6.^18^ This is evident in the limited clinical efficacy observed with anakinra, an IL-1 receptor antagonist, in RA treatment.^18^

Several mechanisms actively promote and regulate inflammation and extracellular matrix degradation, especially in bone and cartilage. The apparent diversity in response to treatment underscores that RA is not a single disease but rather a condition with multiple pathways that can induce autoreactivity and synovial inflammation, resulting in a common clinical presentation.^19^ Recognising the existence of multiple mechanisms contributing to a similar disease expression helps to explain the differences in therapeutic response observed with different targeting agents (**Table 1**).^19^

**Table 1.** Highlights on bDMARDs and tsDMARDs in rheumatoid arthritis.

**Drug**	**Mechanism of Action**	**Adverse Events**	**Indications**
TNFi	Neutralises TNF-α or binds to TNF-α receptors. Increases regulatory T cell frequency	Infections, tuberculosis reactivation, cytopenia, nonmelanoma skin cancer, demyelinating syndromes, congestive heart failure.	Second-line therapy in patients with one unsuccessful csDMARD and poor prognostic factors (autoantibodies, high disease activity, bone erosion) or two unsuccessful csDMARD.
Anti-IL-6	Binds to IL-6 receptors	Infections, lipid abnormalities, anaemia, liver enzyme elevations and insulin resistance.	Use in combination with methotrexate as the first biologic agent in csDMARDs/methotrexate failures. It can be considered the first line in bDMARD monotherapy.
Anti-CD20	Binds to CD20 and induces cytotoxicity against B cells	infections, hepatitis B reactivation, and progressive multifocal leukoencephalopathy	Patients who have failed at least one csDMARD and a bDMARD (antiTNF). First- line bDMARD when there is the history of lymphoproliferative disorder.
CD80/86 Inhibitors	Prevents CD80/86-mediated T cell activation	Infections, tuberculosis reactivation, and leukopenia	Patients who have failed at least one unsuccessful biological or csDMARDs
JAK Inhibitors	Interrupts intracellular signalling cascade that activates immune cells	Gastrointestinal infections, tuberculosis or herpes zoster reactivation, cytopenia, increased risk of venous thromboembolism, and lipid abnormalities.	Patients who have failed two csDMARDs or one csDMARD when there are poor prognostic factors. Consider Risk factors^*^.

## NOVEL DRUGS

### Anti-IL6

Anti-IL-6 drugs target interleukin-6 (IL-6), a proinflammatory cytokine involved in the pathological processes that contribute to the symptoms of RA while playing a crucial role in regulating immune responses and inflammation.^21^ IL-6 affects lymphocyte differentiation and is the main contributor to the response in acute-phase of RA and systemic disease symptoms such as pain, fatigue, depression, anaemia, and cardiovascular disease.^21, 22^ Overall, synovial fluid of affected joints in patients with RA present elevated serum levels of both IL-6 and IL-6 receptors (IL-6R). Their concentrations are associated with disease activity and the extent of joint deterioration.^23,24^ This can incite synovitis and joint damage through migrating neutrophils, osteoclast maturation, and the activation of vascular endothelial growth factor (VEGF), inducing Pannus formation.^23,25^

The main drugs that target IL-6 are IL-6R inhibitors, namely tocilizumab (TCZ) and sarilumab, respectively.^26,27^ IL-6 receptor inhibitors bind to IL-6 receptors, preventing IL-6 from binding to these receptors and initiating downstream signalling. The proinflammatory pathway mediated by IL-6 starts with the interaction of the cytokine with its receptor, which subsequently leads to the formation of a complex of IL-6, IL-6R, and two transmembrane glycoproteins, enabling the activation of Janus kinases (JAKs), leading to the activation of transcription 3 (STAT-3), a molecule that drives a cascade of events that elicit the expression of proinflammatory genes, contributing to the pathogenesis of RA.^22,26,27^

These drugs have been proven effective when combined and as monotherapy, while their efficacy in both forms has been demonstrated to be similar, including being effective in preventing radiologic progression.^28–34^ Multiple studies have supported the safety profile of this drug, and the main adverse effects encountered were cytopenia and infections, with no major long-term effects emerging from real-world data.^35,36^

Yip et al. (2021) highlighted that IL-6 inhibitors can be considered a rational choice for treating RA, especially in patients with comorbidities, high baseline IL-6 levels, or intolerance to other treatments.^37^ As per the European Alliance of Associations for Rheumatology (EULAR) recommendations, IL-6 inhibitors are indicated after failure of two csDMARDs or one csDMARD when there are poor prognostic factors.^38^ The latest guidelines have also been changed to agree with the usage of a second anti-IL6 drug after the failure of the first one, which is now on the same level of recommendation as changing to a different class of bDMARD. It can be considered a first-line treatment if used as monotherapy for situations such as MTX intolerances.^38^ In comparison, both the American College of Rheumatology (ACR) and the Brazilian Society of Rheumatology (BSR) agree that Anti-IL6 drugs can be used as first-line treatment alongside any bDMARD, excluding rituximab in patients who have failed 2 csDMARDs.^39,40^

### Anti-TNF

TNF-α antagonist drugs can be divided into neutralising monoclonal antibodies (infliximab, adalimumab, golimumab), antibody fragments (certolizumab pegol), and soluble TNF receptor constructs (etanercept).^41^ TNF-α is a central cytokine to the inflammatory cascade, modulating the immune response and affecting many aspects of cellular and humoral immunity. Synovium and synovial fluid of patients with RA present elevated TNF-α levels. Due to its influence on various cells in the synovial membrane, it induces local inflammation and pannus formation, which further leads to cartilage and bone damage.^42^

These drugs prevent cellular functions involved in the inflammatory cascade by inhibiting TNF from inducing TNF receptor-mediated processes. TNF antagonists bind to TNF and, therefore, inhibits its binding to TNF receptors in other cells, and they may also induce direct effects on TNF-acting cells, such as apoptosis and cytokine suppression.^43^

TNF inhibitors (TNFi) have been proven to be effective when combined or as monotherapy, being more successful when combined with MTX, when compared to MTX or bDMARD monotherapy.^44–48^ Anti-TNF agents have beneficial effects on preventing disease progression at the bone level, enabling a substantial change in RA treatment.^49^ All TNF-α inhibitors combined with MTX show sustained clinical efficacy and prevent radiographic progression.^50,51^ However, serious infections are reported in patients treated with anti-TNF, such as tuberculosis reactivation, upper respiratory tract infection, and nasopharyngitis.^52^

The EULAR and BSR do not place TNFi above other bDMARDs as an alternative treatment for patients who failed two csDMARDs or one csDMARD when there are poor prognostic factors. According to ACR guidelines, TNFi is preferable to other bDMARDs and should be a first-line treatment after double csDMARD failure.^39^ The guidelines recommend changing to a drug with a different mechanism of action in cases of primary TNFi failure. Moreover, the BSR and EULAR agree on choosing a second anti-TNF drug in cases of secondary TNFi failure.^38–40^

It is important to note that up to 40% of all RA patients treated with Anti-TNF drugs fail to respond adequately to these agents or lose responsiveness over time, which might support the ACR decision.^53^ A meta-analysis of randomised controlled trials comparing responses to different bDMARDs in patients who failed TNF inhibitors concluded that tocilizumab was the best alternative in these patients, followed by Rituximab, Abatacept, and tofacitinib.^54^

### Anti-CD20

CD20 is a hydrophobic transmembrane protein located on mature B lymphocytes. These cells are present in synovial tissue in RA patients and are responsible for antibody production and regulating the initiation of the cell cycle and differentiation.^55,56^ These cells activate phagocytes and complement while providing costimulatory signals to autoreactive T cells, producing cytokine that further activates inflammatory cells.^55^

In 2006, the first anti-CD20 drug, Rituximab (RTX), was approved by the FDA and acts by binding to CD20 in B cells and inducing cytotoxicity against B cells, causing their depletion and apoptosis.^57^ RTX is an effective treatment with long-lasting effects in most patients with RA.^58–60^ It is essential to note that studies have shown RTX is effective in patients with active and long-established RA who had an inadequate response to 1 or more TNFi therapies.^61–64^

Importantly, the most commonly reported safety challenges included de novo infections and reactivation of chronic infections. Due to lack of evidence for or against RTX’s teratogenic effect, it is recommended that women refrain from exposing themselves to RTX at least 6 months before conception.^65^

RTX is the drug that most agrees with the EULAR, ACR, and BSR guidelines. It is indicated for patients who have contraindications or intolerance to the use of TNF inhibitors, a history of lymphoproliferative disorder, or after failure of at least one TNF inhibitor (third-line therapy).^38–40,66,67^

### CD80/86 Inhibitors

The diminution of B-cell activation is achieved through the inhibition of costimulatory molecules, namely CD80/86. These molecules, which reside on the plasma membrane of antigen-presenting cells, engage in ligand-receptor interactions with CD28, a receptor on the surface of CD4+ T cells. This interaction delivers a crucial costimulatory signal, thereby activating T cells. The consequence of this activation process encompasses not only T cell proliferation but also the production of cytokines, contributing to a comprehensive and orchestrated immune response.^68,69^

Abatacept (ABA), the main representative of this drug class, prevents transmission of costimulatory signals from APCs to T cells, and subsequent T cell activation, mediated by CD80/86.^26,68^ ABA has been approved for treating RA in patients after failure of two csDMARDs or one csDMARD when there are poor prognostic factors or after TNFi failure, respectively, in EULAR/BSR and ACR.^38–40^ ABA, in combination with MTX, is generally well tolerated and effective in patients with RA and has been proven to prevent radiographic progression of the disease.^70–74^ Although there have been reports of serious infections, which means it is contraindicated in patients with uncontrolled active infections, many studies found its safety acceptable as it did not differ from other bDMARDs.^75–77^

### Janus Kinase (JAK) Inhibitors

Janus kinases (JAKs) are a group of intracellular tyrosine kinases essential in the pathogenesis of inflammatory diseases for its participation in signalling of numerous cytokines involved in the process.^50^ JAK signal transducers and activators of transcription (STAT) proteins are in synovial tissue and synovial cells. JAK inhibitors interrupt the JAK-STAT intracellular signalling cascade that activates immune cells.^78^

JAK inhibitors are the newest drugs available for RA treatment. Tofacitinib, Upadacitinib, and baricitinib are the drugs of this class that have been approved for use in certain regions. Tofacitinib, a first-generation and nonselective inhibitor, was approved for clinical use in over 80 countries. Baricitinib is also a first-generation but more selective inhibitor generated by modifying the structure of tofacitinib.^79–81^

Their efficacy in improving clinical signs and symptoms and inhibiting the progression of structural joint damage has been documented in clinical trials.^82–85^ The safety of JAK inhibitors is not completely clear in regard to cardiovascular and malignant tumour risk factors, which means that when recommending RA treatment, the risk stratification needs to be highlighted before prescribing these drugs.^86–88^ Their safety for patients without risk factors has shown to be tolerable and approved in multiple countries.^77, 89^

Usage of JAK inhibitor for individuals older than 65 years should consider previous or current smoking, diabetes, obesity, hypertension, current or previous malignant tumour (other than nonmelanoma skin cancer), and risk factors for thromboembolic events.^38^ The main adverse effects of these drugs are infections; however, although the incidence of upper respiratory tract, lower respiratory tract, and urinary tract infections is higher than that in the general population, the incidence is similar to other bDMARDs.^90^

The EULAR recommends JAKi for patients who have failed two csDMARDs or one csDMARD when there are poor prognostic factors. However, it suggests that TNFi may be preferable, especially in patients with high-risk factors for malignancies, thromboembolic events, and cardiovascular events, as the safety profile for these groups of people has not yet been approved.^38^ However, after risk considerations and stratification, it can be considered a first option for patients who must go through b/tsDMARD monotherapy alongside IL-6R inhibitors.^34^ BSR places JAKi as a second-line treatment along with other bDMARDs after careful consideration of the risks, while ACR takes those risks further into account and specifically notes that the patient must fail a TNFi or be intolerant to TNFi to be prescribed JAKi.^39, 40^

## DISCUSSION

In this review, we propose to compare the two main RA guidelines, EULAR and ACR (**Figure 1**), and the recommendations made by the Brazilian Society of Rheumatology about the new strategies of treatment for RA.^38–40^ Current evidence-based guidelines recommend that patients with two csDMARDs or one csDMARD who have failed and have poor prognostic factors.^38–40^ Should initiate treatment with biologics combined with csDMARDs. Monotherapy should be reserved for situations of csDMARD intolerance to improve outcomes, which include radiological damage, symptom control, function, and health-related quality of life in patients with moderate to severe RA.^36,86^

A meta-analysis by Pierreisnard et al. (2013) concluded that biologics combined with MTX are more effective than MTX alone in patients with RA, especially those who do not respond to MTX. However, all biologics showed approximately the same efficacy during the first year of treatment in MTX-naive patients. However, approximately 30% of patients in clinical practice are treated with bDMARDs in monotherapy.^92^ This might be explained by the results found by Amaral et al.^93^ in a cross-sectional study that showed that 20.8% of patients with RA experience MTX intolerance, while a review by Nalwa et al. (2023) compiling the available literature on MTX intolerance in RA reported that the rates of intolerance to MTX range from 11% to 50.5%, with adherence and persistence to the drug being highly variable and with recurrent poor adherence.^95–98^

The EULAR recommends that if a bDMARD or tsDMARD has failed, it should be considered continuing treatment with another bDMARD or a tsDMARD. If one TNF- or IL-6 receptor inhibitor therapy has failed, patients can receive a drug with another mode of action or a second TNFi or IL-6 receptor inhibitor^38^ The superiority of TNFi to non-TNFi drugs needs to be clarified, as some studies report no significant differences in outcomes between TNFi drugs.^98^ Overall, compared with Anti-IL6, higher effectiveness was found for TCZ in mono or combined therapy for patients using csDMARDs and in patients who were bDMARD naive,^99^ as well as increased disease improvement and drug adherence,^100^ supporting the decisions by EULAR and BSR.^38,40^ In patients who failed TNFi, it is not clear which other bDMARD should be the first choice, though some studies proved that switching to RTX and anti-IL-6 were better options than a second TNFi, mainly in cases of primary failure to a TNFi.^101,102^ TCZ was more efficient in this scenario than abatacept and RTX.^103^ At the same time, another study compared the effectiveness of ABA vs TCZ in TNFi-experienced patients and concluded that the patients showed substantial improvement in clinical disease activity. Similar outcomes were observed for both treatment cohort.^104^ This evidence was considered not strong enough by the guidelines to propose changing to a different drug class after the first Anti-TNF drug failure.^38–40^

Anti-IL6 and ABA can be found alongside one another in all guidelines and are used as second-line after TNFi only in ACR, as mentioned.^38–40^ A review by Ogata et al. concluded that TCZ displayed a favourable efficacy in many RA patients, including DMARD naïve patients and patients who failed to respond to csDMARDs or TNFi.^35^ Among its effects, TCZ has been found to improve the quality of life and physical function of people in treatment and symptoms like sleep and fatigue.^105–107^ While inflammation markers, such as PCR, rapidly decrease serum levels, clinical findings are gradually alleviated.^108^ Sarilumab has demonstrated similar tolerability and safety results in clinical trials tocilizumab.^109^ Sarilumab broad efficacy across all RA subtypes has been supported by a set of RCTs, showing a clear superiority over adalimumab in its use as monotherapy in MTX-intolerant subjects.^109–110^ Interestingly, RTX represents the third line of treatment, as its usage is suggested after at least one bDMARD or TNFi failure.

JAKi causes concerns in the guidelines due to the doubts related to the risks of malignancies and thromboembolic and cardiovascular events. The EULAR and BSR place this drug class along with other bDMARDs after cardiovascular risk stratification, but ACR suggests the usage of at least one TNFi of any class before prescribing JAKi.^38–40^ In 2019, JAKi were considered to be at a similar effectiveness and safety level as bDMARDs.^111^ However, the data from ORAL-Surveillance trial, which included patients with RA>50 years of age with cardiovascular risk factors, found more major adverse cardiovascular events (MACEs) and higher malignancy rates related to tofacitinib compared with TNFi, a change in this recommendation was required.^84^ This review has certain limitations, mainly because it was not performed as a systematic review with predefined search criteria; therefore, some relevant studies may have yet to be included. The subjectivity involved in this narrative synthesis may result in a selective presentation of evidence, influencing the overall perspective on the efficacy and safety of biologics in RA treatment. The heterogeneity in study designs and patient populations across various trials exploring biologics in RA can complicate the synthesis of findings and limit the generalisability of conclusions. Nevertheless, the studies presented cover the biologics currently available for treating RA.

The advent of biologics has significantly reshaped the therapeutic landscape for RA, providing substantial clinical benefits for many patients. Evidence-based guidelines endorse the initiation of biologics, either as monotherapy or in combination with csDMARDs. Moreover, the diverse landscape of biological therapies presents clinicians with various options, each demonstrating distinct efficacy and safety profiles. While TNFi is often prioritised, this study reveals nuances in treatment responses based on patient characteristics and previous treatment experiences. The investigation into JAK inhibitors introduces valuable insights, indicating their frequent use following csDMARDs and comparable effectiveness to bDMARDs in clinical practice.

Further research and clinical evidence are imperative to refine treatment strategies and optimise outcomes for RA patients. Simultaneously, efforts are directed toward unravelling gaps in understanding disease patterns across various phases. Although not all patients are responsive to these novel drugs, ongoing research focuses on optimising the use of established therapeutic approaches.

## CONFLICT OF INTEREST

The authors declare no conflict of interest.

## FUNDING

This review did not receive a specific grant from any funding agency in the public, commercial, or not-for-profit sectors.

## DATA AVAILABILITY

All data revised during this study are included in this published article.

## COMPLIANCE WITH ETHICAL STANDARDS

Patients were not involved in this study.

## FUNDING

No funding was applied for this research.

## AUTHOR CONTRIBUTIONS

Conceptualisation: MMPN and CEMR. Analysis: MMPN, AA, ILPN, MSPm ATAM, JFC and CEMR. Writing/original draft: MMPN, AA, ATAM, JFC and CEMR. Writing/review and editing: All authors reviewed the manuscript.
